# Global burden of metabolic-associated fatty liver disease among women of childbearing age: Trends from 1990 to 2021 and projections to 2040

**DOI:** 10.1371/journal.pone.0326244

**Published:** 2025-06-17

**Authors:** Jiaxing Li, Qihui Hu, Jiajie Leng, Baoyong Zhou, Cong Chen, Yaoyue Hu, Bingdi Chao, Zhenhao Huang, Zhenrui Cao, Zhongling An, Jixing Wang, Dingheng Hu, Rui Tao

**Affiliations:** 1 Department of Hepatobiliary Surgery, Bishan Hospital of Chongqing Medical University, Chongqing, China; 2 Department of Cardiothoracic Surgery, The First Affiliated Hospital of Chongqing Medical University, Chongqing, China; 3 School of Public Health, Chongqing Medical University, Chongqing, China; 4 Department of Hepatobiliary Surgery, The First Affiliated Hospital of Chongqing Medical University, Chongqing, China; Kazan State Medical University: Kazanskij gosudarstvennyj medicinskij universitet Ministerstva zdravoohranenia Rossijskoj Federacii, RUSSIAN FEDERATION

## Abstract

**Background:**

Metabolic-associated fatty liver disease (MAFLD) is a growing global health concern, particularly among women of childbearing age (WCBA). We aimed to analyze the global burden of MAFLD among WCBA from 1990 to 2021 and project trends to 2040.

**Methods:**

Data on incidence, prevalence, deaths, and disability-adjusted life years (DALYs) were extracted from the Global Burden of Disease Study 2021. Joinpoint regression and decomposition analysis were used to assess historical trends, and Bayesian Age-Period-Cohort (BAPC) modeling projected future burdens.

**Results:**

From 1990 to 2021, the age-standardized rate (ASR) of MAFLD incidence and prevalence among WCBA increased globally (EAPC = 0.76 and 0.71, respectively). China showed declining trends in deaths (EAPC = −2.63) and DALYs (EAPC = −2.62). By 2040, BAPC modeling predicts a continued rise in global incidence and prevalence, with regional disparities in mortality. Population growth was the primary driver of the global increase in MAFLD incidence, accounting for 63.38% of the rise.

**Conclusion:**

MAFLD imposes a significant burden on WCBA globally, with socioeconomic disparities driving regional variations. Targeted interventions addressing obesogenic environments and healthcare inequities are urgently needed.

## Introduction

Metabolic-associated fatty liver disease (MAFLD) is a liver condition unrelated to alcohol consumption, characterized by the accumulation of fat in the liver without other identifiable causes of liver damage. MAFLD is a chronic progressive liver disease closely associated with insulin resistance and genetic susceptibility. It is estimated that approximately 25% of the global population suffers from MAFLD, making it a major contributor to cirrhosis and hepatocellular carcinoma [1]. Not only is MAFLD the most common cause of chronic liver disease, but it has also been widely recognized as a key factor exacerbating the burden of chronic liver diseases worldwide [2]. Among various potential risk factors, overweight and obesity are particularly significant correlates of MAFLD [3]. Given the global trends in obesity and its related metabolic syndromes, it is anticipated that the burden of MAFLD will further intensify in the future; thus, there is an urgent need for increased attention and research on this issue [4].

One of the United Nations Sustainable Development Goals aims to reduce global maternal mortality rates with a specific target set for 2030: reducing maternal deaths to fewer than 70 per 100,000 live births globally [5]. The prevalence of MAFLD among WCBA has nearly tripled over the past decade. Furthermore, MAFLD has been independently associated with complications such as hypertension during pregnancy, postpartum hemorrhage and preterm birth [6]. However, comprehensive analyses of MAFLD burden among WCBA across socioeconomic regions and its association with adverse maternal health outcomes remain limited, particularly in low-resource settings. This reflects insufficient focus on this particular demographic group which may hinder efforts toward achieving relevant targets within sustainable development goals. The World Health Organization (WHO) defines WCBA as women aged 15–49 years [7].

To address the limitations of existing research and enhance global understanding of MAFLD epidemiology, this study utilized data from the Global Burden of Disease (GBD) 2021 to provide a comprehensive and updated evaluation of the disease’s impact and trends. The objectives of this study were threefold: (1) To conduct a descriptive epidemiological analysis of MAFLD globally, within five sociodemographic index(SDI) regions, and across 204 countries and territories; (2) To perform trend analysis and demographic-epidemiological decomposition to elucidate the factors influencing these trends; (3) To forecast the global burden of MAFLD through 2040, offering a forward-looking perspective on the projected trajectory of the disease.

## Methods

### Data sources

MAFLD burden estimates (incidence, prevalence, deaths, DALYs) for women aged 15–49 years were extracted from the GBD 2021 database (http://ghdx.healthdata.org/)[8]. Existing literature provides detailed descriptions of the methodologies, data sources, and modeling techniques employed in GBD research [9–11]. Age-standardized rates (ASRs) for the incidence, prevalence, deaths, and Disability-Adjusted Life Years (DALYs) were calculated per 100,000 population using the GBD standard population. Uncertainty intervals (UIs) were derived via 1,000 Monte Carlo simulations [12–14].

### Statistical analysis

Joinpoint regression analysis was performed using Joinpoint Trend Analysis Software (Version 5.0.2; National Cancer Institute) to identify inflection points in ASR trends. Bayesian Age-Period-Cohort (BAPC) modeling was implemented in R (version 4.3.1) with the BAPC package, using 10,000 Markov chain Monte Carlo iterations for projections to 2040.

### Sociodemographic index

The Sociodemographic Index (SDI) quantifies social development levels across countries or regions through an amalgamation of income, education, and fertility status. SDI categorizes nations into five regions: low, low-middle, middle, high-middle, and high [15].

### Trend analysis

The study introduces the Estimated Annual Percentage Change (EAPC) along with its 95% confidence interval (CI) to illustrate changes in the burden of MAFLD from 1990 to 2021. The calculation of EAPC is based on a log-linear regression model: ln (ASR) = α + βx, where x represents calendar years; thus, EAPC = (exp(β)-1) *100%. The corresponding 95% CI is derived from the standard error of the model [16].

Furthermore, using Joinpoint Trend Analysis Software (2022), we decompose overall trends into multiple sub-trends by calculating annual percentage changes (APC) and average annual percentage changes (AAPC) at each inflection point [17]. APC and its 95% CI assess the intensity of local epidemiological trends; AAPC serves as a comprehensive indicator for measuring trends in MAFLD burden calculated as a weighted average of APCs [18,19].

### Decomposition analysis

We utilize decomposition analysis to explore driving factors behind changes in global incidence, prevalence, deaths, and DALYs associated with MAFLD from 1990 to 2021. Decomposition analysis facilitates assessment of three factors—population growth, aging demographics, and epidemiological shifts—impacting MAFLD burden [20].

### Predictive analysis

We opted to employ Bayesian Age-Period-Cohort (BAPC) modeling to forecast future burdens related to MAFLD across China globally and within five different SDI regions. The BAPC model assumes that future trends in MAFLD burden will follow historical patterns observed between 1990 and 2021, extrapolated through age-specific, period-specific, and cohort-specific effects. Key assumptions include: Demographic continuity: Population growth rates and age structures remain consistent with GBD 2021 projections; Epidemiological stability: No major interventions will disrupt current MAFLD progression trends; Linear extrapolation of risk factors: Obesity and metabolic syndrome drivers continue to rise at rates observed in 2010–2021. This modeling approach generates evidence-based projections to inform resource allocation and policy development for MAFLD mitigation [21,22].

### Ethics approval statement

The study data were initially derived from the Global Burden of Disease study, Institute for Health Metrics and Evaluation, which abides by relevant guidelines and regulations. Data from the GBD study are freely available to researchers and policymakers.

## Results

### Descriptive analysis of MAFLD burden among WCBA

Globally, there has been a marked increase of case number in the incidence, prevalence, deaths, and DALYs associated with MAFLD among WCBA from 1990 to 2021. In 2021, the ASRs of incidence and prevalence were significantly higher than those recorded in 1990, as illustrated in S1 Table. In China, the trends in MAFLD incidence and prevalence mirrored global patterns during this period; however, trends for deaths and DALYs exhibited an inverse correlation. Across the five SDI regions, both incidence and prevalence aligned with global trends. Conversely, while high-middle and low-middle SDI regions reflected similar patterns for deaths and DALYs as observed globally, other SDI regions experienced an increase in absolute numbers but a decline in ASR.

In 2021, Qatar exhibited the highest age-standardized incidence rate (ASIR) of MAFLD among WCBA (1,863.58 per 100,000; 95% UI: 1,301.74 to 2,526.21), followed by Kuwait (1,856.66) and Egypt (1,817.94) (S2 Table). Turkmenistan reported the highest mortality rate (2.40 per 100,000; 95% UI: 1.19 to 4.47), followed by the Republic of Moldova (2.11) and the Russian Federation (1.68).

### Trends of MAFLD burden among WCBA by using EAPC at global and regional levels

Between 1990 and 2021, the global landscape of MAFLD exhibited a consistent annual increase in incidence, prevalence, and DALYs, with EAPC value of 0.76 (95% CI: 0.72 to 0.79), 0.71 (95% CI: 0.66 to 0.77) and 0.04 (95% CI: −0.05 to 0.12). Deaths remained stable, reflected by an EAPC value of 0 (95% CI: −0.09 to 0.09) as detailed in S1 Table. In contrast, China’s EAPC for MAFLD incidence and prevalence were recorded at 0.56 (95% CI: 0.41 to 0.72) and 0.45 (95% CI: 0.24 to 0.66) respectively; meanwhile, deaths and DALY experienced declines of −2.63 (95% CI: −2.95 to −2.31) and −2.62 (95% CI: −2.94 to −2.29) respectively—all lower than the global averages.

Examining the SDI regions, an upward trend in both the incidence and prevalence of MAFLD among WCBA was observed from 1990 to 2021. However, the EAPC values for deaths and DALYs increased in low-middle and high-middle SDI regions, contrasting with a decline noted in other areas. Notably, the high-middle SDI region experienced the most significant changes in EAPC for both deaths and DALYs, with increases of 1.04 (95% CI: 0.68 to 1.41) and 1.10 (95% CI: 0.74 to 1.46), respectively. Meanwhile, the high SDI region exhibited the largest absolute increases in EAPC for incidence and prevalence at the value of 1.17 (95% CI: 1.12 to 1.23) and 1.28 (95% CI: 1.23 to 1.33), respectively.

### Joinpoint regression analysis on local trends in MAFLD burden among WCBA

**Fig 1:**
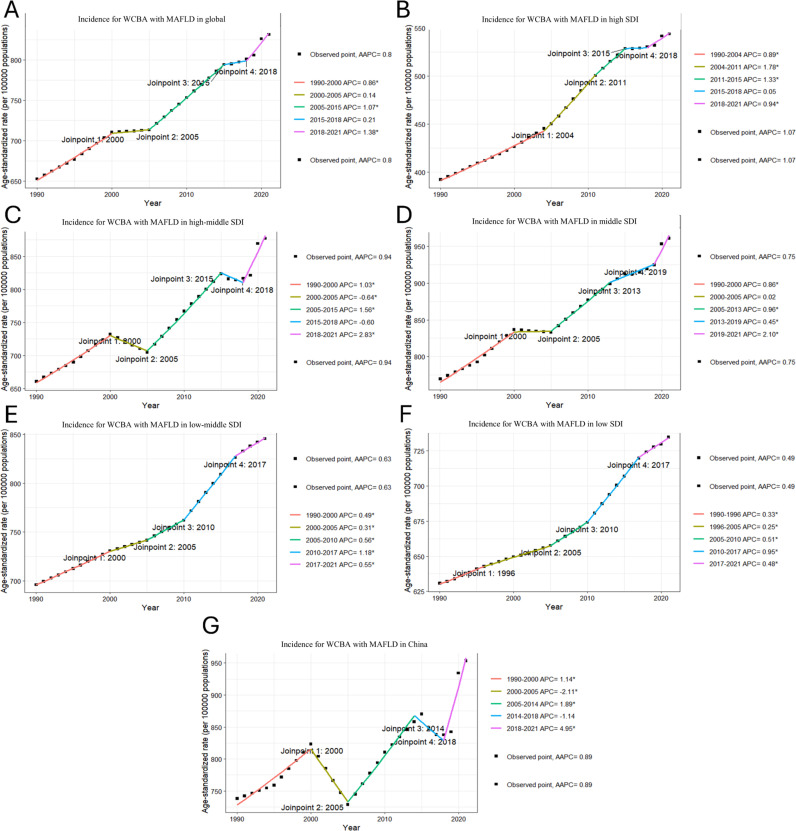
Joinpoint regression analysis on the ASR of incidence with MAFLD among WCBA. (A) in global, (B)in high SDI, (C)in high-middle SDI, (D) in middle SDI, (E)in low-middle SDI, (F) in low SDI, (G) in China.

**Fig 2:**
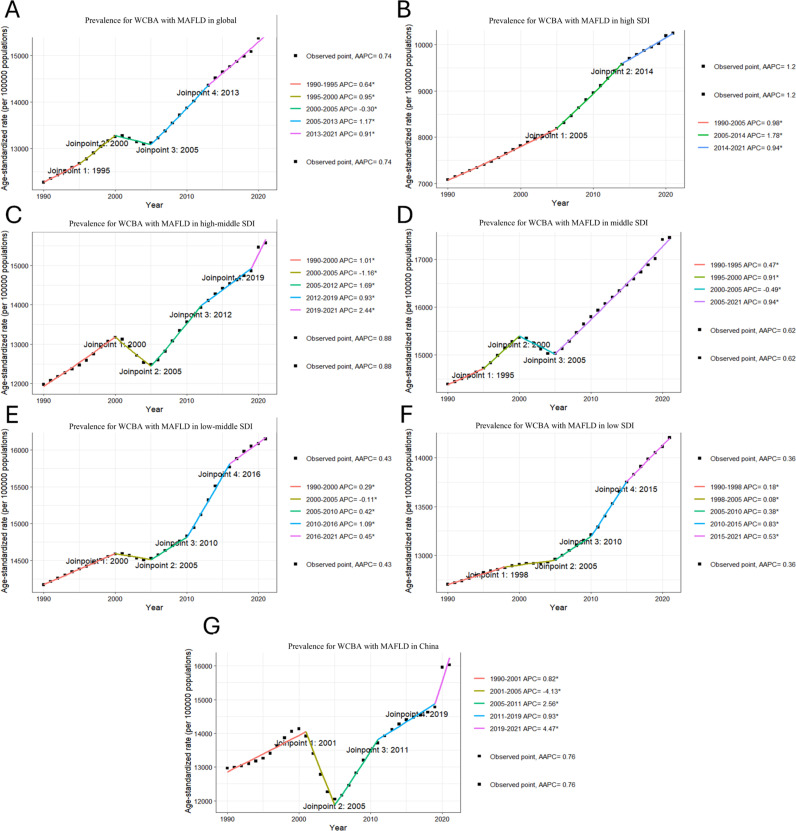
Joinpoint regression analysis on the ASR of prevalence with MAFLD among WCBA. (A) in global, (B)in high SDI, (C)in high-middle SDI, (D) in middle SDI, (E)in low-middle SDI, (F) in low SDI, (G) in China.

The Joinpoint regression analysis of the burden of MAFLD among WCBA is illustrated. Between 1990 and 2021, both the global ASR of incidence (AAPC = 0.8) and prevalence (AAPC = 0.74) exhibited a consistent upward trend in. In contrast, the ASR for global deaths (AAPC = 0.08) remained largely stable over this period. However, the ASR for global DALYs demonstrated a fluctuating pattern, characterized by an initial peak followed by a decline and subsequent resurgence. In China, trends in ASR for incidence (AAPC = 1.14) and prevalence (AAPC = 0.76) mirrored each other; both initially increased before declining to a peak, then decreasing again before rising once more. Conversely, ASR for deaths showed a continuous downward trajectory (AAPC = −2.31). The pattern observed for DALYs was marked by a sustained decline followed by a slight increase and ultimately another decrease (AAPC = −2.24). Across five distinct SDI regions, the ASRs for incidence and prevalence generally displayed an upward fluctuation as depicted in Fig 1 and 2. Notably divergent patterns were observed concerning the ASR of deaths: with the exception of the high-middle SDI region—which experienced an initial rise followed by a decrease to its peak— the ASR of deaths across other regions remained relatively stable throughout this timeframe. Regarding DALYs, significant variations emerged among the five SDI strata: The middle SDI region witnessed a decline to its lowest point prior to experiencing an increase; conversely, the low-middle SDI region saw an initial rise leading to its peak before entering into decline. The remaining SDI regions exhibited patterns characterized by fluctuating increases in DALYs.

### Decomposition analysis on MAFLD burden in 2021

**Fig 3:**
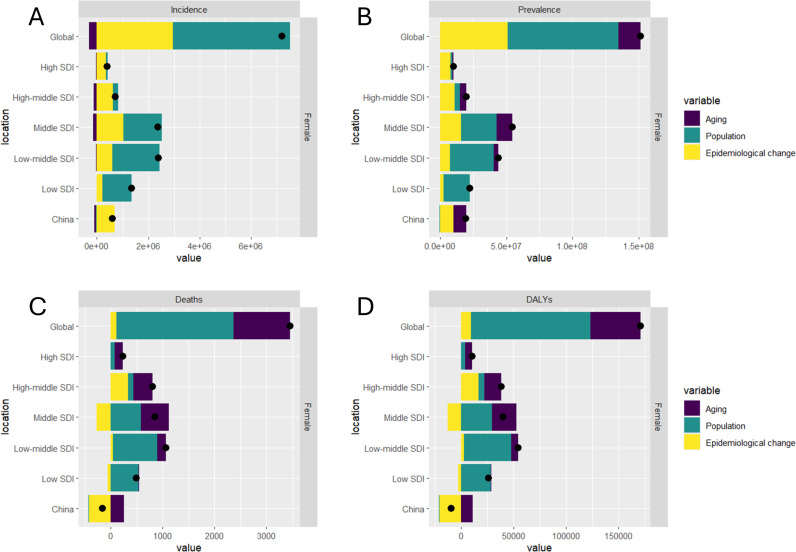
Decomposition analysis on the ASR of incidence, prevalence, deaths, and DALYs with MAFLD among WCBA in global, 5 SDI regions and China. (A) incidence, (B) prevalence, (C)deaths, (D)DALYs.

In our study, we conducted a comprehensive decomposition analysis to evaluate the incidence, prevalence, deaths, and DALYs associated with MAFLD in China, globally, and across five SDI regions. This analysis aimed to elucidate the individual contributions of population growth, aging, and epidemiological shifts to the disease burden over the study period. The findings are illustrated in Fig 3. The black dots represent the aggregate effect of all three factors; positive values indicate an increase while negative values denote a decrease in the respective metric.

On a global scale, we observed a significant rise in MAFLD incidence, prevalence, deaths, and DALYs, with population growth emerging as the predominant influencing factor. Globally, population expansion was identified as the primary driver behind the increased burden of MAFLD: contributing 63.38%, 55.15%, 65.08%, and 66.68% to the rises in incidence, prevalence, deaths, and DALYs respectively from 1990 to 2021 (Fig 3). In contrast to global trends, epidemiological changes had a particularly pronounced impact in China—accounting for increases of 254.58%, 216.48%, 52.05%, and 115.35% in MAFLD incidence, prevalence, deaths, and DALYs respectively during this same period. Among the five SDI regions analyzed, the low-middle SDI region exhibited marked increases in MAFLD incidence, prevalence, and deaths (Fig 3A, 3C, and 3D). Notably, in terms of prevalence, the middle SDI region experienced the most substantial increase (Fig 3B).

### Predictive analysis on MAFLD trend to 2040

**Fig 4:**
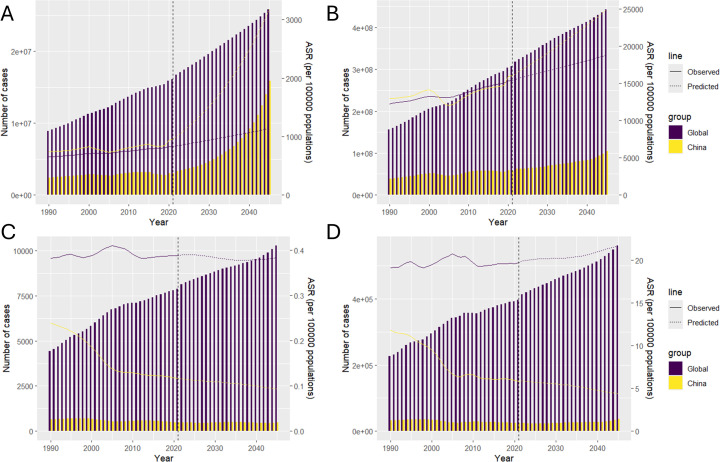
Predictive analysis on the cases number of incidence, prevalence, deaths, and DALYs with MAFLD among WCBA to 2040. (A) incidence, (B) prevalence, (C)deaths, (D)DALYs.

Utilizing the BAPC modeling approach, we projected the incidence, prevalence, deaths, and DALYs associated with MAFLD from 2021 to 2040, as illustrated in Fig 4. Our analysis indicated a consistent upward trend in both the incidence and prevalence of MAFLD in China and globally (Fig 4A and 4B). Regarding deaths and DALYs, we anticipate a significant global increase; however, these metrics are expected to remain relatively stable within China (Fig 4C and 4D). This divergence suggests distinct trajectories for disease burden across different regions, underscoring the necessity for tailored public health strategies to address these variations.

## Discussion

The rising burden of MAFLD amongWCBA is closely linked to the global epidemic of metabolic syndrome. Obesity, a central driver of MAFLD, promotes hepatic lipid accumulation and fibrosis through lipotoxicity, insulin resistance, and chronic inflammation [1]. Our study found that high SDI regions exhibited the largest increase in MAFLD incidence (EAPC = 1.17), directly associated with urbanization-driven sedentary lifestyles, and high-calorie diets in these areas [2]. Pregnancy-specific metabolic changes further exacerbate this risk: elevated estrogen and placental lactogen levels enhance insulin resistance, stimulating hepatic lipogenesis [23]. Additionally, pregnant women with MAFLD face significantly higher risks of gestational hypertension and preterm birth (OR = 1.8) [6], a mechanism linked to intensified hepatic lipid peroxidation and oxidative stress [23].

This study reveals marked regional disparities in MAFLD burden. Middle Eastern countries had the highest global incidence rates (ASIR > 1,800 per 100,000), consistent with Golabi et al.’s report of a tripled MAFLD prevalence in the Middle East and North Africa over the past decade, primarily attributed to rising obesity rates and insufficient public health interventions [2]. However, unlike Golabi’s findings, our analysis identified Eastern European nations with the highest MAFLD -related mortality (ASMR > 2 per 100,000), likely due to inequitable healthcare resource distribution and delayed diagnoses. For instance, Turkmenistan’s primary care system screens less than 30% of at-risk populations for MAFLD, leading to late-stage diagnoses at cirrhosis [24].

The decline in MAFLD mortality in China (EAPC = −2.63) is likely attributable to the nationwide implementation of the 2024 Chinese Guidelines for Metabolic Dysfunction-Associated Fatty Liver Disease, which mandate non-invasive fibrosis screening during prenatal care [25]. Conversely, high SDI regions continue to experience rising MAFLD incidence despite abundant healthcare resources, suggesting that clinical management alone cannot offset cumulative lifestyle risks [3].

Although NAFLD affects approximately 25% of the general population [1], WCBA faces unique risk amplification. Pregnancy is not only an independent risk factor for MAFLD but may also influence offspring metabolic health via epigenetic mechanisms, such as DNA methylation [26]. A prospective cohort study demonstrated that MAFLD during pregnancy increases postpartum hemorrhage risk by 1.8-fold [6]. Our predictive model further projects a 34% global rise in MAFLD -related DALYs among WCBA by 2040 without intervention, underscoring the urgency of integrating MAFLD into maternal health surveillance systems.

This study has several limitations. First, the data from the GBD database used in this study is based on the GBD 2021 update. However, there have been significant advancements in MAFLD treatment. In 2024, the FDA approved the first – ever MALFD therapy. Resmetirom (Rezdiffra) is a once – daily oral selective THR – β agonist. It is indicated for adults with non – cirrhotic MAFLD and moderate – to – advanced liver fibrosis [27]. The absence of these recent developments in the model may compromise the accuracy of future projections. Second, the analysis did not account for unmeasured confounders such as dietary patterns and genetic factors, which play a critical role in MAFLD progression [28]. Future research should integrate multi – omics data to refine risk stratification models [28]. Third, data sparsity in low-resource settings may have reduced the accuracy of our regional projections. For instance, mortality estimates in Eastern Europe relied on incomplete vital registration systems, potentially underestimating MAFLD-related deaths. Future studies should integrate alternative data sources to address these gaps.

## Conclusion

MAFLD represents a significant and escalating global health challenge, particularly in regions such as China. Our research identifies WCBA as a demographic especially vulnerable to this condition and underscores the socioeconomic disparities observed across various SDI regions. This study provides, for the first time, a comprehensive overview of the incidence, prevalence, deaths, and DALYs associated with MAFLD among WCBA worldwide. The analysis spans five SDI regions and encompasses 204 countries and territories from 1990 to 2021, along with projections for 2040. Between 1990 and 2021, there has been a general increase in the global burden of MAFLD among WCBA. We have noted a significant rise in incidence, prevalence, deaths, and DALYs attributed to MAFLD on a global scale; population growth is identified as the primary contributing factor. Projections indicate that addressing MAFLD must be recognized as an urgent priority within global health agendas. Immediate and coordinated actions across multiple sectors are essential to mitigate the increasing prevalence of this disease. Such measures should include implementing policies aimed at reducing obesogenic environments, enhancing community education initiatives, establishing rigorous screening programs, and improving medical infrastructure—particularly in resource-constrained settings.

## Supporting information

S1 Table The number, ASR, and EAPC of incidence, prevalence, deaths, and DALYs of MAFLD among WCBA in 1990 and 2021 in global, China, and 5 SDI. (XLSX)

S2 Table The number and ASR of incidence, prevalence, deaths, and DALYs of MAFLD among WCBA in 1990 and 2021 in global 204 countries. (XLSX)
